# Survey datasets on women participation in green jobs in the construction industry

**DOI:** 10.1016/j.dib.2018.02.009

**Published:** 2018-02-09

**Authors:** Adedeji O. Afolabi, Rapheal A. Ojelabi, Patience F. Tunji-Olayeni, Olabosipo I. Fagbenle, Timothy O. Mosaku

**Affiliations:** Department of Building Technology, Covenant University, Nigeria

## Abstract

The unique qualities of women can make them bearers of solutions towards achieving sustainability and dealing with the dangers attributed to climate change. The attitudinal study utilized a questionnaire instrument to obtain perception of female construction professionals. By using a well-structured questionnaire, data was obtained on women participating in green jobs in the construction Industry. Descriptive statistics is performed on the collected data and presented in tables and mean scores (MS). In addition, inferential statistics of categorical regression was performed on the data to determine the level of influence (beta factor) the identified barriers had on the level of participation in green jobs. Barriers and the socio-economic benefits which can guide policies and actions on attracting, retaining and exploring the capabilities of women in green jobs can be obtained from the survey data when analyzed.

**Specifications table**

**Table:** 

Subject area	*Environmental Science*
More specific subject area	*Green jobs*
Type of data	*Tables, Figures and Text files*
How data was acquired	*Field Survey*
Data format	*Raw*
Experimental factors	*Purposive sampling of women construction professionals in diverse fields in the construction industry*
Experimental features	*Sample selection of the perception of women construction professionals on participation, barriers and socio-economic benefits in green jobs in the construction industry*
Data source location	*Lagos, Nigeria*
Data accessibility	*All the data are in this data article*

**Value of the data**

• The questionnaire instrument is compact and can be adapted or modified for studies in other climes, thereby comparing the results from under-developed, developing and developed countries.
• The data provided the descriptive statistics for the selected sample for measuring the level of participation of women compared to men in green jobs in the construction industry.
• The data when completely analyzed can provide insight into the obstacles hindering the career advancement of women in green jobs in the construction industry, while the socio-economic benefits of engaging women in green jobs if well harness can help the environment and the construction industry.
• An understanding of the barriers and socio-economic benefits can guide policy makers and construction industry stakeholders on ways to tackle the shortage of women participation in the construction industry.
• The data can increase the awareness of women and the girl child on the distinct features of green jobs in contrast to other jobs available in the construction industry in general.

## 1 Data

**Fig. 1:**
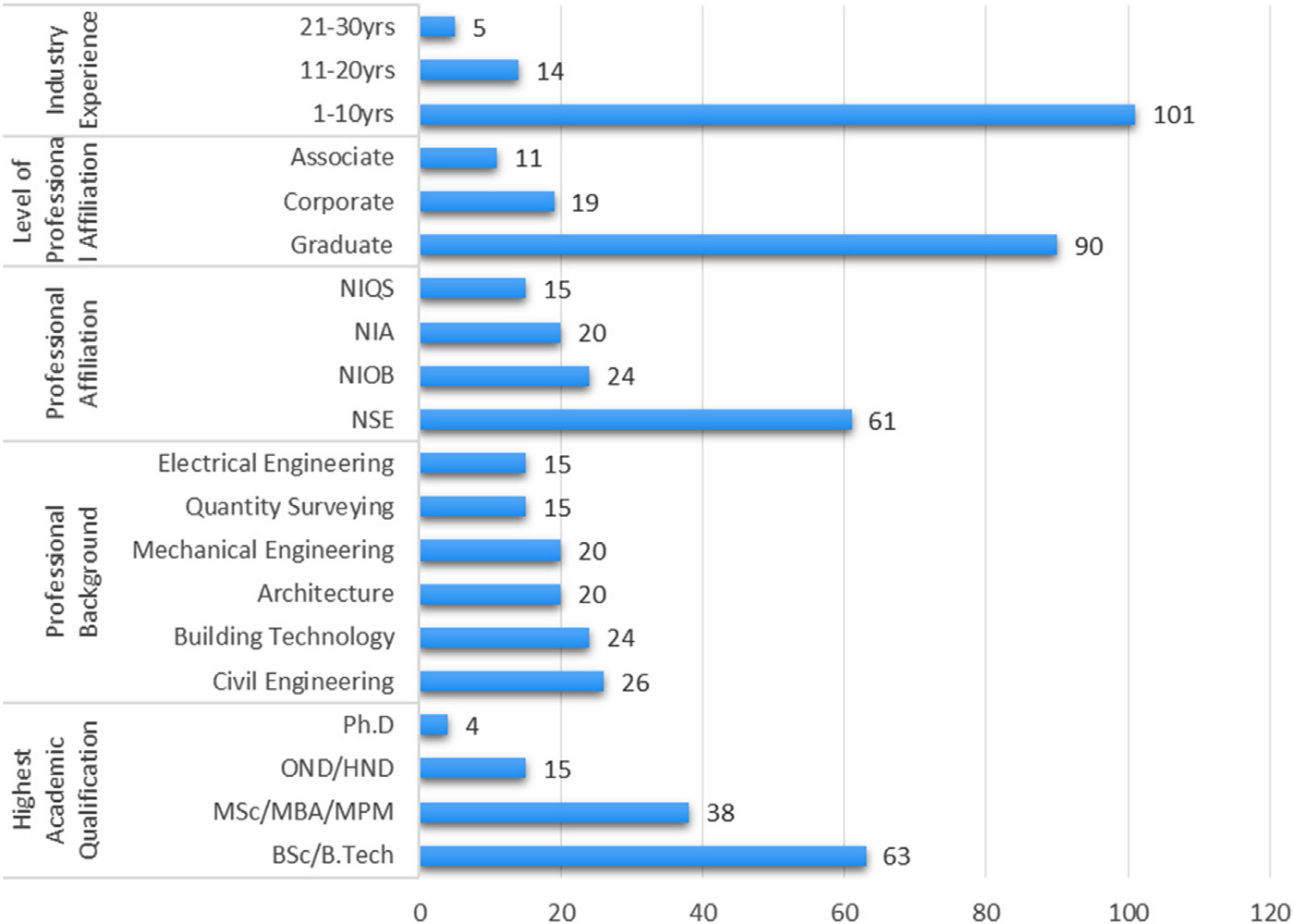
**:** Summarized overview of the background information of the women construction professionals.

**Fig. 2:**
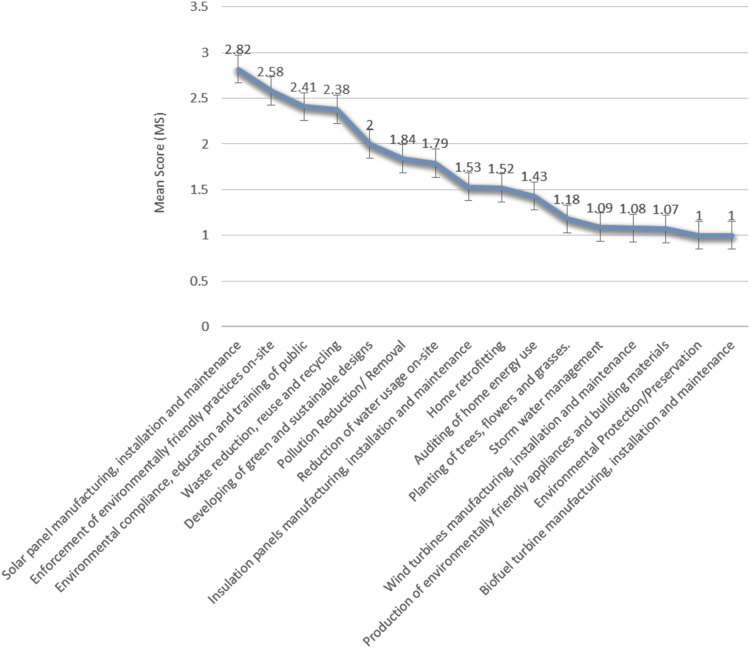
Level of participation of women in green jobs in the construction industry.

The data instrument of a well-structured questionnaire was administered to one hundred and twenty (120) women construction professionals in Lagos State, Nigeria. The demographic characteristics of the female construction professionals is shown in Fig. 1 and Table 1. The designed data instrument guided the contents of the data which helped to determine the level of women participation in green jobs in the construction industry. The data analysis can reveal the inhibiting factors to the participation of women in green jobs. An understanding of the data can help in harnessing the socio-economic benefits to the girl child, women, the environment and the construction industry as a whole. Research questions can be posed, which in turn can lead to inferential statistics, which when interpreted can inform the development of policies and strategic actions on women inclusion in the construction industry. As such, the categorical regression was used to determine the level of influence the identified barriers had on the level of participation in green jobs. The data revealed areas that are peculiar to provision of green jobs in the construction sector such as Solar panel manufacturing, installation and maintenance, Enforcement of environmentally friendly practices on-site, Environmental compliance, education and training of public, Waste reduction, reuse and recycling, Developing of green and sustainable designs, Pollution Reduction/ Removal, Reduction of water usage on-site, Insulation panels manufacturing, installation and maintenance, Home retrofitting, Auditing of home energy use, Planting of trees, flowers and grasses, Storm water management, Wind turbines manufacturing, installation and maintenance, Production of environmentally friendly appliances and building materials, Environmental Protection/Preservation, Biofuel turbine manufacturing, installation and maintenance. The uniqueness of this data is its focus on green jobs which is an area of employment generation and deals with issues of climate change and sustainability. Finally, the data can be used as a comparative study with other under-developed and developed countries. The data can be assessed as Supplementary data 1 and the questionnaire can be assessed as Supplementary data 2 (Table 2, Table 3, Table 4) and Fig. 2.

**Table 1:** Summary of the Total score of the background information of the sample.

	**Highest academic qualification**	**Professional background**	**Professional affiliation**	**Level of professional qualification**	**Industry experience**
Mean	2.26	3.39	2.98	3.07	1.20
Std. Error of Mean	.065	.151	.107	.045	.045
Median	2.00	4.00	4.00	3.00	1.00
Mode	2	4	4	3	1
Std. Deviation	.716	1.657	1.177	.498	.495
Variance	.513	2.744	1.386	.248	.245
Skewness	.132	.030	-.579	.144	2.497
Std. Error of Skewness	.221	.221	.221	.221	.221
Kurtosis	-.177	-1.227	-1.267	1.054	5.493
Std. Error of Kurtosis	.438	.438	.438	.438	.438
Range	3	5	3	2	2
Minimum	1	1	1	2	1
Maximum	4	6	4	4	3

**Table 2:** Barriers to women participation in green jobs in the construction industry.

**Barriers**	**Minimum**	**Maximum**	**Mean**	**Std. deviation**
Male dominance of green jobs	4	5	4.83	.374
Discrimination	4	5	4.75	.435
Low level of green job training	4	5	4.67	.473
Lack of experience	4	5	4.58	.495
Lack of role models in these fields	4	5	4.42	.495
Biased recruitment	4	5	4.27	.444
Inadequate skills sets required	4	5	4.19	.395
Cultural and traditional factors	3	5	4.02	.518
Position at work	3	5	3.95	.672
Reluctance to accept women skills	3	5	3.77	.618
Limited green job opportunities	3	5	3.67	.653
Harsh working conditions/ environment	3	5	3.60	.666
Stressful nature of work	3	5	3.53	.660
Low interest from women	3	5	3.43	.576
Low career growth/ progression	2	5	3.35	.694
Low investment in green works	2	5	3.27	.786
Low pay in green jobs	2	5	3.27	.670
Sexual harassment	2	5	3.18	.635

**Table 3:** Socio-economic benefits of women participation in green jobs in the construction industry.

**Socio-economic benefits**	**Minimum**	**Maximum**	**Mean**	**Std. deviation**
Increased awareness on sustainable solutions	4	5	4.88	.332
Increased education attainment among females	4	5	4.79	.408
Increased roles for women	4	5	4.71	.456
Sustainable growth and development	4	5	4.63	.486
Economic growth for nations	4	5	4.53	.501
Improved ecosystem	4	5	4.39	.490
Better household energy consumption	4	5	4.28	.453
Creation of more green jobs	4	5	4.13	.341
Improved family health	4	5	4.08	.278
Harnessing women strength and skills	3	5	4.01	.399
Increased participation of women in other sectors	3	5	3.92	.495
Increased energy efficiency and security	3	5	3.83	.560
Cost effective solutions	3	4	3.66	.476
Increased green energy market	3	4	3.58	.496
Cheaper renewable energy solutions	3	4	3.53	.501
Improved human welfare	2	4	3.45	.646
Increase organizations and countries’ commitment to cut GHG emissions	2	4	3.35	.630
Increased investment in green economy	2	4	3.27	.730
Reduction of environmental pollution	2	4	3.18	.698
Reduction in production and use of pollutants	2	4	3.06	.802
Reduction of sick building syndrome	2	4	2.89	.696
Better waste generation and management	2	4	2.77	.590
Better air quality	2	4	2.68	.505

**Table 4:** Categorical Regression of Barriers influencing the level of participation in green construction.

**Barriers**	**Standardized coefficients**		df	F	Sig.
	**Beta**	**Bootstrap (1000) Estimate of std. error**			
Male dominance of green jobs	.003	.114	1	.001	.979
Stressful nature of work	.179	.295	2	.371	.691
Biased recruitment	.007	.126	1	.003	.955
Discrimination	.060	.119	1	.250	.618
Low level of green job training	-.076	.118	1	.413	.522
Inadequate skills sets required	-.044	.136	1	.103	.749
Sexual harassment	.345	.272	3	1.607	.193
Low interest from women	-.176	.302	2	.341	.712
Position at work	-.016	.160	1	.011	.919
Harsh working conditions/ environment	-.402	.294	2	1.873	.160
Low career growth/ progression	.429	.200	3	4.625	.005**
Lack of experience	-.038	.137	1	.078	.781
Low investment in green works	-.214	.223	3	.925	.432
Low pay in green jobs	-.332	.287	2	1.342	.267
Cultural and traditional factors	-.051	.154	1	.112	.739
Reluctance to accept women skills	-.204	.234	2	.761	.470
Limited green job opportunities	.512	.210	2	5.963	.004**
Lack of role models in these fields	-.177	.120	1	2.183	.143
Dependent Variable: Professional Background					

## 2 Experimental design, materials and methods

The data collected was built on previous research conducted on women participation in the construction industry and the areas of green jobs that appear in the construction industry. Details on other researched works on the subject can be found in [1], [2], [3], [4], [5], [6], [7], [8], [9], [10], [11], [12], [13], [14]. The population is a summation of all women in the construction industry in Nigeria. For this study, the commercial nerve centre in Nigeria was selected, which is Lagos State. Lagos State is an economic hub of Nigeria and highly developed, with high concentration of construction professionals, high volume of state of the art completed and ongoing construction projects and high applications in the areas of green construction. The state has been classified as a Mega city and is presently arming itself with necessary buildings and infrastructure to cope with its new status. The women used for this study fall into the categories of construction professionals in the fields of architecture, building technology, quantity surveying, mechanical, civil and electrical engineering. The sample survey is based on a subset of women in the construction industry in Lagos State. Similar researches that used field survey to obtain their data can also be found in [15], [16], [17], [18], [19], [20], [21], [22], [23], [24], [25]. A survey research design was conducted on the identified sample. The respondents were chosen randomly using a purposive sampling method due to the characteristics of the sample. This method was used due to the easy access of the respondents to the researcher. The questionnaire is measured using a five point Likert scale. A total of 180 questionnaires were distributed to women in the construction industry in Lagos State. A total of 120 completed questionnaires were returned which were adequately scrutinized of errors and omissions, this represented a 66.7% return rate. For future research, the difference between women and their male counterpart's participation in green jobs can be explored. Specific to the practical experiences of women in green jobs, the interview technique could be used to obtain raw data for analysis.

## References

[1] Adogbo K.J., Ibrahim A.D., Ibrahim Y.M. (2015). Development of a framework for attracting and retaining women in construction practice. J. Constr. Dev. Ctries..

[2] Afolabi A., Emeghe I., Oyeyipo O., Ojelabi R. (2016). Professionals' preference for migrant craftsmen in Lagos state. Mediterr. J. Soc.. Sci..

[3] Agapiou A. (2002). Perceptions of gender roles and attitudes toward work among male and female operatives in the Scottish construction industry. Constr. Manag. Econ..

[4] Bruyere S., Filiberto D. (2013). The green economy and job creation: inclusion of people with disabilities. Int. J. Green Econ..

[5] Errázuriz O. (2010). Special Policy Dialogue: the Role of Women in Countries in Special Situations. Dialogues at the Economic and Social Council: achieving Gender Equality, Women Empowerment and Strengthening Development Cooperation.

[6] Gurjao S. (2008). Inclusivity: The Changing Role of Women in the Construction Workforce.

[7] Oyeyipo O.O., Odusami K.T., Ojelabi R.A., Afolabi A.O. (2016). Factors affecting contractors' bidding decision for construction projects in Nigeria. J. Constr. Dev. Ctries..

[8] Ogunde A.O., Olaolu O., Afolabi A., Owolabi J. (2017). J. and R. Ojelabi, Challenges confronting construction project management system for sustainable construction in developing countries: professionals perspectives (a case study of Nigeria). J. Build. Perform..

[9] Kumar B.R. (2013). Gender discrimination among construction workers with reference to Vijayawada. J. Sociol. Soc. Work.

[10] L. Amusan, P. Tunji-Olayeni, A. Afolabi, I. Omuh, R. Ojelabi, A. Oluwatobi, Remodularising technical institutions towards quality manpower delivery in construction sector in Nigeria, in: Proceedings of the 10th Annual International Technology, Education and Development Conference, 7th–9th March, Valencia, Spain, 2016.

[11] Lingard H., Francis V., Gale A.W., Davidson M.J. (2006). Work-life balance in construction: promoting diversity. Managing Diversity in the Construction Sector.

[12] Tunji-Olayeni P., Emetere M.E., Afolabi A. (2017). Multilayer perceptron network model for construction material procurement in fast developing cities. Int. J. Civ. Eng. Technol..

[13] Whittock M. (2002). Women's experiences of non-traditional employment: is equality in this area a possibility?. Constr. Manag. Econ..

[14] A. Afolabi, M. Dada, An evaluation of the factors affecting housing and urban development projects in Lagos State. Paper presented at the Proceeding of CIB W107 International Conference on Construction in Developing Countries and its Contribution to Sustainable Development, University of Lagos, Lagos, Nigeria, 28th–30th January, 2014.

[15] Bishop S.A., Owoloko E.A., Okagbue H.I., Oguntunde P.E., Odetunmibi O.A., Opanuga A.A. (2017). Survey Datasets on the externalizing behaviors of primary school pupils and secondary school students in some selected schools in Ogun State, Nigeria. Data Brief.

[16] Dimara E., Manganari E., Skuras D. (2017). Survey data on factors influencing participation in towel reuse programs. Data Brief.

[17] Canesi R., Marella G. (2017). Residential construction cost: an Italian survey. Data Brief.

[18] Vuong Q.H. (2016). Survey data on entrepreneurs' subjective plan and perceptions of the likelihood of success. Data Brief.

[19] Sanfo S., Fonta M.W., Boubacar I., Lamers P.A.J. (2016). Survey data on key climate and environmental drivers of farmers' migration in Burkina Faso, West Africa. Data Brief.

[20] Jridi O., Nouri F.Z. (2015). Survey of socio-economic and contextual factors of households' energy consumption. Data Brief.

[21] Okagbue H.I., Opanuga A.A., Oguntunde P.E., Ugwoke P.O. (2017). Random number datasets generated from statistical analysis of randomly sampled GSM recharge cards. Data Brief.

[22] Giannoccaro G. (2017). Survey data of stated farmer's preferences and willingness to supply straw. Data Brief.

[23] Ibrahim M.R. (2017). A dataset of housing market and self-attitudes towards housing location choices in Alexandria Egypt. Data Brief.

[24] Akinyemiju T., Moore J.X. (2016). Data on burden of comorbidities in the United States and Medicaid expansion. Data Brief.

[25] Vuong Q.-H., Nguyen T.-K. (2016). Data on Vietnamese patients' financial burdens and risk of destitution. Data Brief.

