# Supplementary material for: Survey datasets on women participation in green jobs in the construction industry
Source: Data Brief. 2018 Feb 9;17:856–62. doi: 10.1016/j.dib.2018.02.009 (PMC5834649; doi:10.1016/j.dib.2018.02.009)
Supplement: Supplementary file 2 — Supplementary material [file mmc2.docx]

**QUESTIONNAIRE**

**WOMEN PARTICIPATION IN GREEN JOBS.**

**Dear Respondent,**

This research questionnaire aims at collecting data for purely academic purpose. The information obtained through this medium will be treated absolutely as confidential.

Regards,

Researcher.

**SECTION A: BACKGROUND INFORMATION.**

Please, kindly indicate as relevant**☒**

1. Highest academic qualification attained: OND/HND **☐**^1^ BSc/B.Tech **☐**^2^ MSc/MBA/MPM **☐** ^3^ Ph.D. **☐** ^4^ Others (please specify) ______________ **☐** ^5^
2. Professional Background: Architecture **☐**^1^ Builder **☐**^2^  Quantity Surveying **☐** ^3^ Civil Engineering**☐**^4^ Mechanical Engineering**☐**^5^ Electrical Engineering**☐**^4^
3. Professional Affiliation: NIA **☐**^1^ NIOB**☐**^2^  NIQS **☐** ^3^ NSE**☐**^4^
4. Level of Professional Qualification: Licentiate **☐**^1^ Associate**☐**^2^  Graduate **☐** ^3^ Corporate**☐**^4^ Fellow **☐**^5^
5. Industry experience of the respondent: 1-10yrs **☐**^1^ 11-20yrs **☐**^2^ 21-30yrs **☐**^3^

31-40yrs **☐**^4^ 41-50yrs **☐**^5^ above 50yrs **☐**^6^

**SECTION B: IDENTIFYING AREAS OF WOMEN PARTICIPATION IN GREEN JOBS.**

1. Please kindly indicate the frequency of your participation in these areas of green jobs using this criteria **5 = Very High, 4 = High, 3 = Moderate, 2 = Low, 1 = Not at all**

| S/No | **AREAS** | **5** | **4** | **3** | **2** | **1** |
| --- | --- | --- | --- | --- | --- | --- |
| 1 | Solar panel manufacturing, installation and maintenance | **☐** | **☐** | **☐** | **☐** | **☐** |
| 2 | Wind turbines manufacturing, installation and maintenance | **☐** | **☐** | **☐** | **☐** | **☐** |
| 3 | Biofuel turbine manufacturing, installation and maintenance | **☐** | **☐** | **☐** | **☐** | **☐** |
| 4 | Auditing of home energy use | **☐** | **☐** | **☐** | **☐** | **☐** |
| 5 | Home retrofitting | **☐** | **☐** | **☐** | **☐** | **☐** |
| 6 | Pollution Reduction/ Removal | **☐** | **☐** | **☐** | **☐** | **☐** |
| 7 | Storm water management | **☐** | **☐** | **☐** | **☐** | **☐** |
| 8 | Environmental compliance, education and training of public | **☐** | **☐** | **☐** | **☐** | **☐** |
| 9 | Developing of green and sustainable designs | **☐** | **☐** | **☐** | **☐** | **☐** |
| 10 | Waste reduction, reuse and recycling | **☐** | **☐** | **☐** | **☐** | **☐** |
| 11 | Production of environmentally friendly appliances and building materials | **☐** | **☐** | **☐** | **☐** | **☐** |
| 12 | Enforcement of environmentally friendly practices on-site | **☐** | **☐** | **☐** | **☐** | **☐** |
| 13 | Environmental Protection/Preservation | **☐** | **☐** | **☐** | **☐** | **☐** |
| 14 | Insulation panels manufacturing, installation and maintenance | **☐** | **☐** | **☐** | **☐** | **☐** |
| 15 | Reduction of water usage on-site | **☐** | **☐** | **☐** | **☐** | **☐** |
| 16 | Planting of trees, flowers and grasses. | **☐** | **☐** | **☐** | **☐** | **☐** |

**SECTION C: ASSESSING THE BARRIERS TO WOMEN PARTICIPATION IN GREEN JOBS.**

1. Please kindly indicate the barriers to women participation in green jobs using **5 = Very Significant, 4 = Significant, 3 = Moderately significant, 2 = of Little significance, 1 = Insignificant**

| **S/No.** | **BARRIERS** | **5** | **4** | **3** | **2** | **1** |
| --- | --- | --- | --- | --- | --- | --- |
| 1 | Male dominance of green jobs | **☐** | **☐** | **☐** | **☐** | **☐** |
| 2 | Stressful nature of work | **☐** | **☐** | **☐** | **☐** | **☐** |
| 3 | Biased recruitment | **☐** | **☐** | **☐** | **☐** | **☐** |
| 4 | Discrimination | **☐** | **☐** | **☐** | **☐** | **☐** |
| 5 | Low level of green job training | **☐** | **☐** | **☐** | **☐** | **☐** |
| 6 | Inadequate skills sets required | **☐** | **☐** | **☐** | **☐** | **☐** |
| 7 | Sexual harassment | **☐** | **☐** | **☐** | **☐** | **☐** |
| 8 | Low interest from women | **☐** | **☐** | **☐** | **☐** | **☐** |
| 9 | Position at work | **☐** | **☐** | **☐** | **☐** | **☐** |
| 10 | Harsh working conditions/ environment | **☐** | **☐** | **☐** | **☐** | **☐** |
| 11 | Low career growth/ progression | **☐** | **☐** | **☐** | **☐** | **☐** |
| 12 | Lack of experience | **☐** | **☐** | **☐** | **☐** | **☐** |
| 13 | Low investment in green works | **☐** | **☐** | **☐** | **☐** | **☐** |
| 14 | Low pay in green jobs | **☐** | **☐** | **☐** | **☐** | **☐** |
| 15 | Cultural and traditional factors | **☐** | **☐** | **☐** | **☐** | **☐** |
| 16 | Reluctance to accept women skills | **☐** | **☐** | **☐** | **☐** | **☐** |
| 17 | Limited green job opportunities | **☐** | **☐** | **☐** | **☐** | **☐** |
| 18 | Lack of role models in these fields | **☐** | **☐** | **☐** | **☐** | **☐** |

**SECTION D: EXAMINING THE SOCIO-ECONOMIC BENEFITS OF WOMEN PARTICIPATION IN GREEN JOBS**

1. Please kindly indicate the socio-economic benefits of women participation in green jobs using the criteria **5 = Very Significant, 4 = Significant, 3 = Moderately significant, 2 = of Little significance,**

**1 = Insignificant**

| **S/No.** | **BENEFITS** | **5** | **4** | **3** | **2** | **1** |
| --- | --- | --- | --- | --- | --- | --- |
| 1 | Improved family health | **☐** | **☐** | **☐** | **☐** | **☐** |
| 2 | Improved human welfare | **☐** | **☐** | **☐** | **☐** | **☐** |
| 3 | Increase organizations and countries’ commitment to cut GHG emissions | **☐** | **☐** | **☐** | **☐** | **☐** |
| 4 | Sustainable growth and development | **☐** | **☐** | **☐** | **☐** | **☐** |
| 5 | Harnessing women strength and skills | **☐** | **☐** | **☐** | **☐** | **☐** |
| 6 | Increased education attainment among females | **☐** | **☐** | **☐** | **☐** | **☐** |
| 7 | Increased investment in green economy | **☐** | **☐** | **☐** | **☐** | **☐** |
| 8 | Reduction of sick building syndrome | **☐** | **☐** | **☐** | **☐** | **☐** |
| 9 | Increased participation of women in other sectors | **☐** | **☐** | **☐** | **☐** | **☐** |
| 10 | Reduction of environmental pollution | **☐** | **☐** | **☐** | **☐** | **☐** |
| 11 | Increased awareness on sustainable solutions | **☐** | **☐** | **☐** | **☐** | **☐** |
| 12 | Creation of more green jobs | **☐** | **☐** | **☐** | **☐** | **☐** |
| 13 | Reduction in production and use of pollutants | **☐** | **☐** | **☐** | **☐** | **☐** |
| 14 | Increased roles for women | **☐** | **☐** | **☐** | **☐** | **☐** |
| 15 | Increased energy efficiency and security | **☐** | **☐** | **☐** | **☐** | **☐** |
| 16 | Improved ecosystem | **☐** | **☐** | **☐** | **☐** | **☐** |
| 17 | Better air quality | **☐** | **☐** | **☐** | **☐** | **☐** |
| 18 | Cost effective solutions | **☐** | **☐** | **☐** | **☐** | **☐** |
| 19 | Better waste generation and management | **☐** | **☐** | **☐** | **☐** | **☐** |
| 20 | Better household energy consumption | **☐** | **☐** | **☐** | **☐** | **☐** |
| 21 | Cheaper renewable energy solutions | **☐** | **☐** | **☐** | **☐** | **☐** |
| 22 | Economic growth for nations | **☐** | **☐** | **☐** | **☐** | **☐** |
| 23 | Increased green energy market | **☐** | **☐** | **☐** | **☐** | **☐** |
